# Retrospective Study for the Safer Management for Citizens' Marathon: A Medical Support Perspective

**DOI:** 10.7759/cureus.87424

**Published:** 2025-07-07

**Authors:** Fumihiro Ogawa, Riichiro Nakayama, Yusuke Nakayama, Yuji Yuasa, Tomohiro Kamagata, Kohei Takahashi, Ryosuke Furuya, Shouhei Imaki, Ichiro Takeuchi

**Affiliations:** 1 Emergency Care, Yokohama City University Hospital, Yokohama, JPN; 2 Medicine, Medical Committee for Yokohama Marathon, Yokohama, JPN; 3 Emergency Medicine, Yokohama City Minato Red Cross Hospital, Yokohama, JPN; 4 Emergency Medicine, Keiyu Hospital, Yokohama, JPN; 5 Emergency Medicine, Yokohama Municipal Citizen's Hospital, Yokohama, JPN; 6 Critical Care and Emergency Medicine, National Hospital Organization Yokohama Medical Center, Yokohama, JPN; 7 Emergency and Critical Care Medical Center, Yokohama Municipal Citizen's Hospital, Yokohama, JPN; 8 Advanced Critical Care and Emergency Center, Yokohama City University Medical Center, Yokohama, JPN

**Keywords:** automated external defibrillator aed, basic life support (bls), mass gathering, out-of-hospital cardiac arrest, running related injuries, sports injury and prevention

## Abstract

Background

Increased sports participation, including marathons, necessitates robust medical support due to inherent health risks like cardiac arrest. Effective safety systems for mass-gathering events require early risk prediction, timely medical intervention, and pre-identified transport routes to medical facilities for emergencies.

Objective

This descriptive epidemiological study aimed to analyze medical incidents at the Yokohama Citizen's Marathon since the introduction of its full marathon in 2015. It also sought to describe trends in the evolving medical support system and their associated outcomes.

Methods

A retrospective review of medical records from 2015 to 2024 was conducted. The study specifically focused on the incidence of cardiac arrest, heat stroke, muscle cramps, and other related conditions encountered by runners, alongside an examination of the concurrent changes in the marathon's medical support framework over the study period.

Results

Across 134,946 full marathon participants (147,861 total runners), 4669 medical staff (3.1% ± 0.4% of runners) were deployed. A total of 136 emergency transports (an average annual rate of 0.1% of runners, ranging from 0.05% to 0.2%) occurred, with 27 hospitalizations (an average annual rate of 0.02% of runners, ranging from 0.01% to 0.03%) and three cases of cardiac arrest (an average annual rate of 0.002% of runners, ranging from 0% to 0.004%). The patient presentation ratio (PPR) was 14.76 per 1000 runners annually (ranging from 11.1 to 17.7), while the transport-to-hospital ratio (TTHR) was 0.96 per 100 patient presentations annually (ranging from 0.4 to 2.1).

Discussion

Annual analysis and subsequent updates to medical protocols were found to be associated with a lower patient presentation ratio and a stable transport-to-hospital ratio when compared to data from previous reports. These findings suggest that systematic improvements in both the planning and response phases of the medical support system contributed to observed reductions in emergency incidents during marathon events.

Conclusion

The continuous evaluation and iterative adjustment of medical data are crucial for enhancing safety in large-scale marathon events. While a majority of medical incidents involved minor illnesses, the occurrence of life-threatening emergencies, such as cardiac arrest, profoundly underscores the indispensable need for meticulous planning, robust interdisciplinary collaboration among medical teams, and effective communication strategies. Ongoing, data-driven adjustments to medical protocols and response mechanisms, adapted to varying conditions of mass-gathering events, are essential to ensure the continued safety and well-being of participants in citizen marathons.

## Introduction

Running has grown in popularity since the 1970s, with a surge in participants and events since 2000 due to its health benefits and association with longevity [1]. Marathons and road races, including those in Japan, have become common worldwide and are considered "mass-gathering events" with over 1000 participants at a specific time and place [2, 3]. These events can strain local healthcare systems, especially when dependent on emergency medical services (EMS), potentially compromising community care [4-6]. Therefore, effective medical planning is crucial to address potential injuries, illnesses, and life-threatening emergencies like cardiac arrest (CA) that may occur during these events, and we must provide independent medical support to determine participants' needs without overburdening local EMS or hospitals [7].

While most injuries at such events are minor, highlighting the importance of first aid (FA) stations, some cases involve serious injuries or medical emergencies/conditions [8-10]. In such scenarios, a two-tiered approach is essential: on-site care for minor issues and emergency response for severe cases [6]. Additionally, organizers must be prepared for mass-casualty incidents, requiring robust emergency and disaster response systems [7, 11]. A well-prepared healthcare system reduces the load on EMS and hospitals, maintains care quality for participants, and protects community healthcare capacity.

Mass-gathering events were scaled down in 2020 due to the COVID-19 pandemic but have since resumed. Fluctuations in event organization and participant numbers during this period are relevant as background information for the medical intervention cases analyzed in this study. Despite the health benefits of running, risks such as CA remain, with an incidence of 2.18 per 100,000 runners reported in Japan [12]. Thus, preparedness, including the deployment of mobile automated external defibrillator (AED) units, rapid cardiopulmonary resuscitation (CPR), and efficient transport, is vital. Furthermore, pre-planning for environmental factors, injury prevention, and emergency response routes is essential for ensuring runner safety. Additionally, organizers must be prepared for mass-gathering incidents, requiring robust emergency and disaster response systems, specifically the establishment of a clear command structure, pre-trained medical teams, rapid information dissemination protocols, and coordinated collaboration with nearby medical institutions.

The Yokohama Marathon, introduced as a full marathon in 2015, has grown significantly in popularity. Over the years, its medical support system has evolved to better respond to the diverse health needs of its participants. This evolution includes the strategic deployment of FA stations, temporary clinics, basic life support (BLS) teams, and FR teams equipped with AEDs.

Objectives

This descriptive epidemiological study aimed to characterize the incidence and outcomes of medical incidents, including cardiac arrest, among participants of the Yokohama Marathon; to describe the evolution and components of the medical support system implemented during the Yokohama Marathon events; and to evaluate the observed trends in patient presentation and transport-to-hospital ratios in relation to the evolving medical support strategies, thereby demonstrating the utility of retrospective data analysis for enhancing event safety.

This article was previously posted to the medRxiv preprint server on February 10th, 2025.

## Materials and methods

We retrospectively examined the incidence of injuries or medical emergencies/conditions (e.g., heat stroke, muscle cramp) and cardiac arrest (CA) among participants and citizens during the Yokohama Marathon from 2015 to 2023, using medical records provided by the organizing committee. We also reviewed changes in the medical support system over time. This study employed a descriptive epidemiological approach. Data were summarized using frequencies, percentages, and means with standard deviations where appropriate. Trends over time were observed and presented graphically and numerically. No inferential statistical analyses were performed to determine causal relationships or statistical significance between changes in the medical support system and observed outcomes, as the primary aim of this study was descriptive reporting of trends and operational insights rather than hypothesis testing or establishing causality. Data were collected from the official Yokohama marathon website on January 12, 2024. This also includes data on weather conditions (temperature, humidity) for each event day.

Yokohama marathon course and medical system

**Figure 1:**
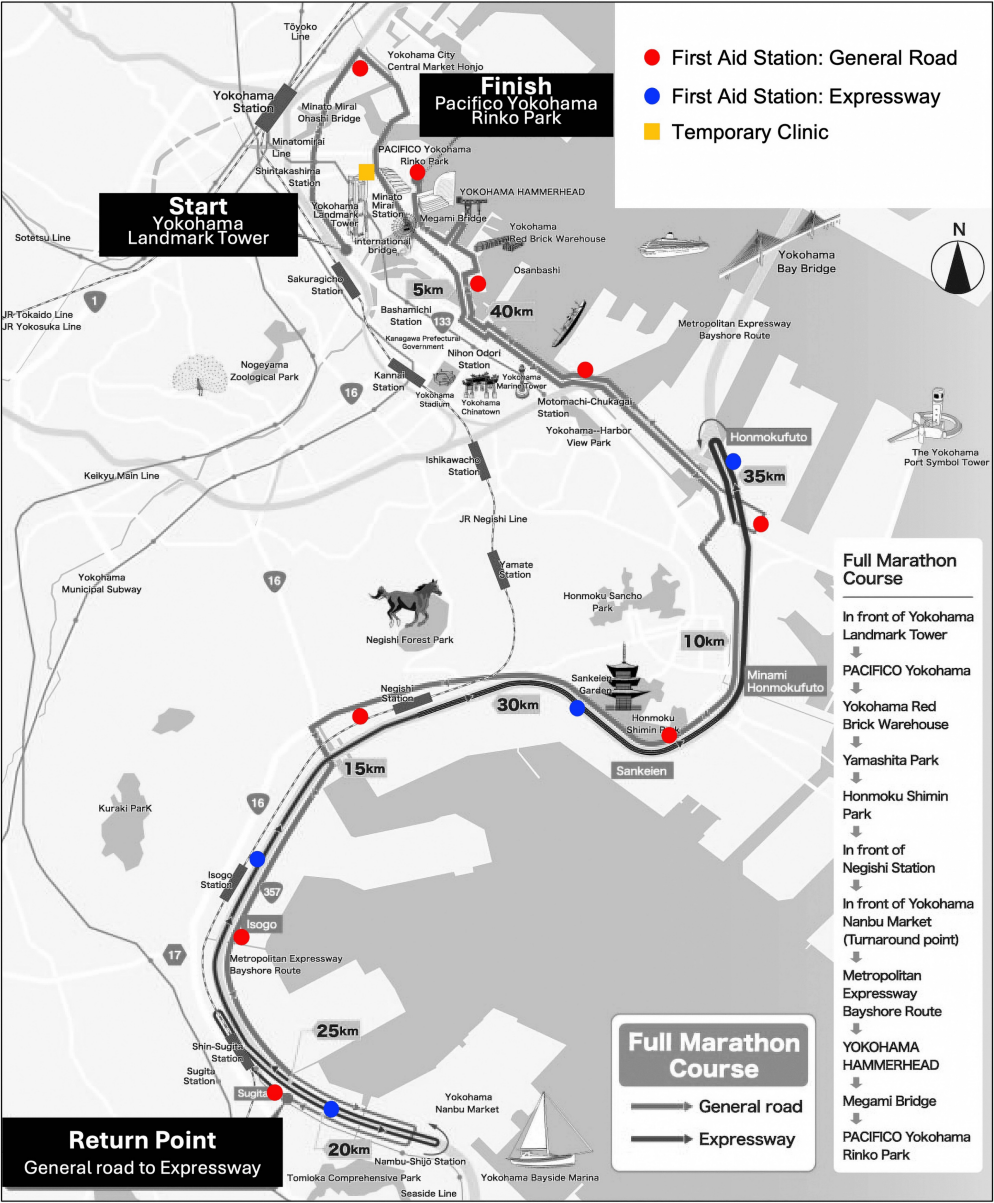
Course map of the Yokohama marathon and medical support system The 42.195 km course runs through the bay area of Yokohama City, Japan, and includes 12 FA stations, one post-finish FA station, and a temporary clinic with BLS teams or FR teams. FA - first aid; BLS - basic life support; FR - first responder

The 42.195 km course runs through the bay area of Yokohama City, Japan, and includes challenging segments (Figure 1). Specifically, a portion of the course, including the 15.5 km mark, features steep inclines and also includes an open highway section with no spectators, which can pose a significant physical burden for runners.

Medical support staff, including doctors, nurses, and paramedics from local institutions, operate under a city-organized team. The system includes 12 FA stations every 3-5 km, one post-finish FA station, and a temporary clinic. These are staffed with medical personnel and equipped for BLS and emergency interventions such as infusion and intubation.

Mobile AED system and first responders

**Figure 2:**
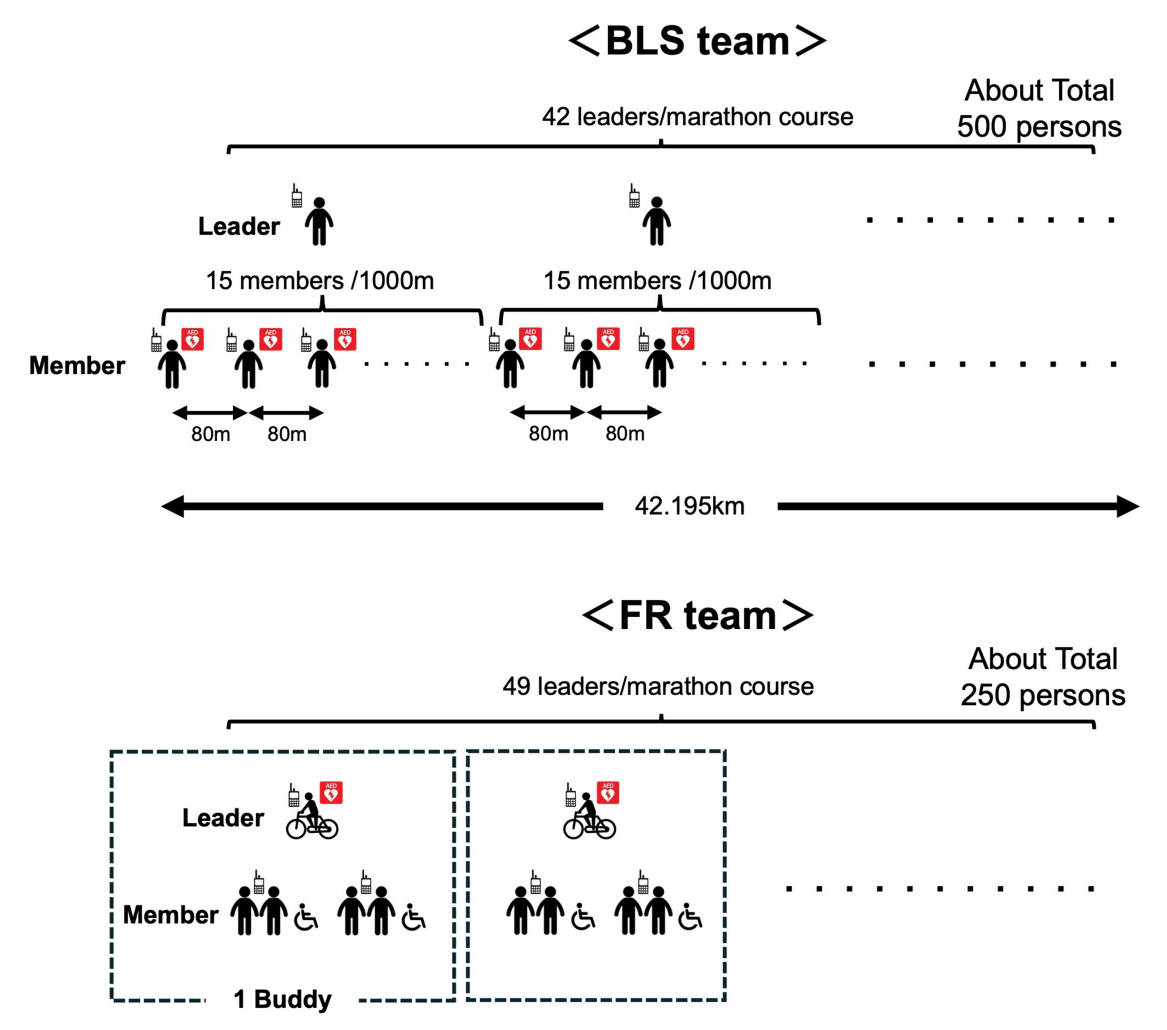
BLS and FR team system Before the COVID-19 pandemic, one BLS team leader was positioned every 1000m along the marathon course (a total of 42 leaders), and a BLS member (approximately 450-500 members) was positioned every 80m. After the COVID-19 pandemic, this is the same theory as the BLS team, but due to personnel allocation, the policy was to place 49 FR team leaders on the marathon course, with each FR team leader assigned four team members (total of five persons), and to operate the team in buddy pairs with bicycle created the rapid mobile AED system to treat runners. BLS - basic life support; FR - first responder; COVID-19 - coronavirus infection disease 2019; AED - automated external defibrator

As shown in Figure 2, from the BLS team before the COVID-19 pandemic (until 2019), to ensure timely AED access to runners experiencing cardiac arrest, one BLS team leader was positioned every 1000 m along the marathon course (a total of 42 leaders) and a BLS member (approximately 450-500 members) was positioned every 80 m. Since 2022, post-pandemic, first responder (FR) teams (a total of 49 teams) with five members each (a team leader assigned four team members), often using bicycles, have fulfilled the same function. This transition from BLS to FR teams was implemented to enhance rapid response capabilities and dynamic coverage across the course, especially in light of evolving operational needs post-COVID-19. While this shift aimed to optimize personnel allocation and improve response efficiency, potential trade-offs, such as changes in the precise number or type of individual responders per area, are acknowledged and are subject to ongoing evaluation.

Volunteers and running doctors

Non-medical volunteers trained in FA/BLS assist in emergencies by reporting to the command center. 'Running Doctors' - medical professionals participating in the race assist runners and activate medical response systems using mobile communication. This initiative is often associated with the Japan Medical Joggers Association (JMJA), which holds the registered trademark for 'Running Doctor' in Japan and promotes such volunteer activities.

Command and control

At the command control center, a doctor acts as the medical director, who is responsible for medical support, and some doctors act as the medical controller between the command center, FA station, and temporary clinic via phone or transceivers at any time. Moreover, approximately 20 paramedics act as dispatchers in the fire departments. In cases of CA, injury, or sickness, the command control center gets some information from bystanders or doctors at the FA place who witness and perform basic telecommunication. The medical control doctor provides advice via phone in the case of an emergency. If an incident occurs on any marathon course at the command center, the medical control doctor and fire department work together to dispatch ambulances to the scene in an appropriate manner and decide the destination in advance to ensure smooth transport of the CA, injured, or sick runner. Through bystander interviews, medical records, and event audits, they create timeline data and medical records.

Calculation of patient presentation ratio and transport-to-hospital ratio

In this study, patient presentation ratio (PPR) and transport-to-hospital ratio (TTHR) were calculated using the number of runners as the denominator. PPR was defined as the number of runners seen at first aid (FA) stations or temporary clinics per 1,000 full marathon participants. TTHR was defined as the number of runners transported to a hospital per 100 patient presentations at FA stations or temporary clinics. Repeat visits by the same runner were counted as separate patient presentations if they occurred at different times or stations, reflecting distinct medical encounters. This approach aligns with common practice in marathon-specific medical research, where the focus is on the health risks and medical needs of active participants subjected to physical exertion. The study focused solely on runners because their unique physical stressors and injury patterns are distinct from those of spectators or volunteers. Data regarding medical incidents among spectators and residents were not systematically collected in a manner comparable to runner data, thus precluding their inclusion in these specific ratios. This distinction is important given the differing types of medical emergencies anticipated between actively participating athletes and the general public in a mass gathering setting.

Statistical analysis

This study adopted a descriptive epidemiological approach, collecting medical record data and event-related data. Data were summarized using frequencies, percentages, means, and standard deviations where appropriate. Trends over time were observed and presented graphically and numerically. No inferential statistical analyses were performed to determine causal relationships or statistical significance between changes in the medical support system and observed outcomes.

## Results

Weather and temperature

**Figure 3:**
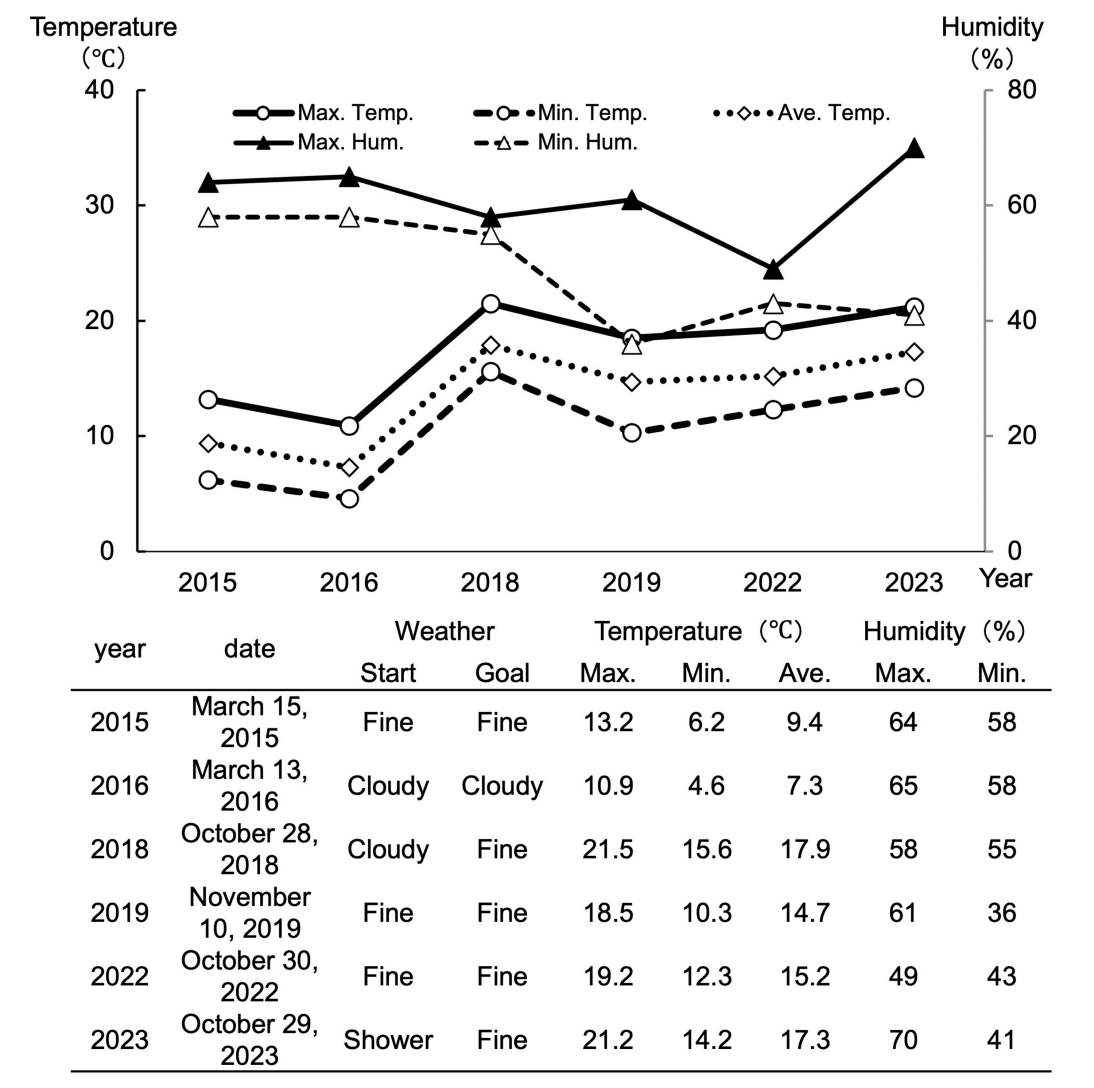
Weather and temperature This figure shows the weather and temperature on the day of the marathon. Temperature (maximum, minimum, average) and humidity (maximum, minimum) are displayed over time.

Figure 3 shows the weather and temperature changes on the days of the marathons. The weather in 2015 and 2016 was cloudy with sunny spells, with maximum temperatures of 13.2 °C and 10.9 °C, respectively, and minimum temperatures of 6.2 °C and 4.6 °C, respectively (average temperatures were 9.4 °C and 7.3 °C, respectively). In 2017, the race was canceled due to poor weather. From 2018 (after the race date shifted to October), maximum temperatures exceeded 20 °C, likely increasing runner strain. Humidity remained relatively unchanged.

Runner and medical staff characteristics

**Figure 4:**
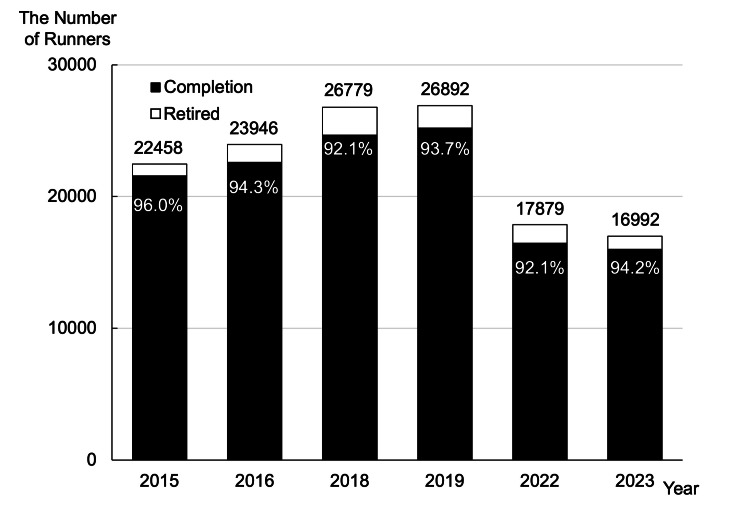
The number of runners and completion ratio This figure shows the annual trends in full marathon participants and completion rates. In 2022, the number of applicants decreased due to COVID-19, but the completion rate did not change significantly.

Of approximately 135,000 full marathon participants (100%) from 2015 to 2023, the participant demographic showed a male predominance, with 105,300 runners (78%) being male. The age distribution was as follows: approximately 810 runners (0.6%) aged under 20 years, approximately 12,555 runners (9.3%) in their 20s, approximately 25,785 runners (19.1%) in their 30s, approximately 44,415 runners (32.9%) in their 40s, approximately 38,880 runners (28.8%) in their 50s, approximately 10,800 runners (8.0%) in their 60s, and approximately 1620 runners (1.2%) aged over 70 years (Figure 4). These percentages represent the overall distribution across the study period. Completion rates were consistently high (92-96%) before and after COVID-19. Post-pandemic races (resumed in 2023) had fewer participants.

**Figure 5:**
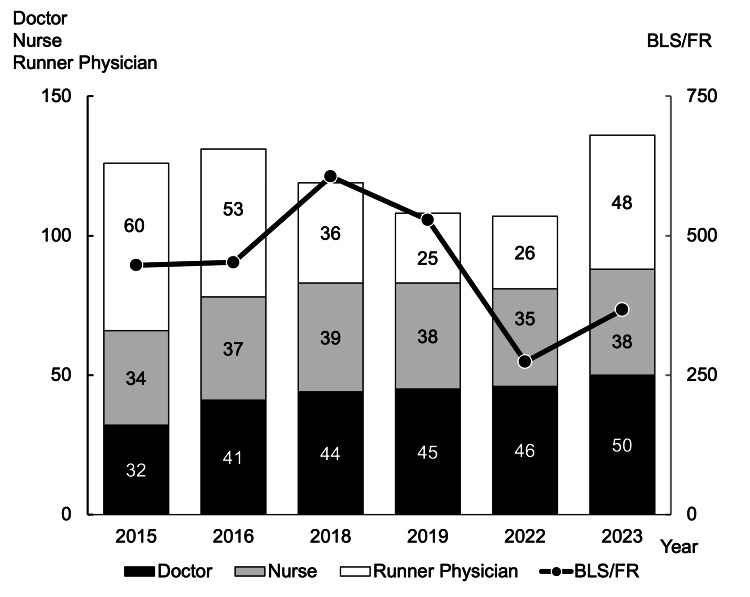
Transition of medical support staff The number of medical staff at first aid stations and temporary clinics supporting the marathon has not changed much. The number of BLS/FR members has decreased after the COVID-19 pandemic. BLS - basic life support; FR - first responder; COVID-19 - coronavirus infection disease 2019; AED - automated external defibrator

In Figure 5, the number of cooperating doctors increased over time, while BLS teams transitioned to FR teams after the COVID-19 pandemic, reducing personnel slightly because of preventing the spread of COVID-19.

Medical cases and interventions

**Figure 6:**
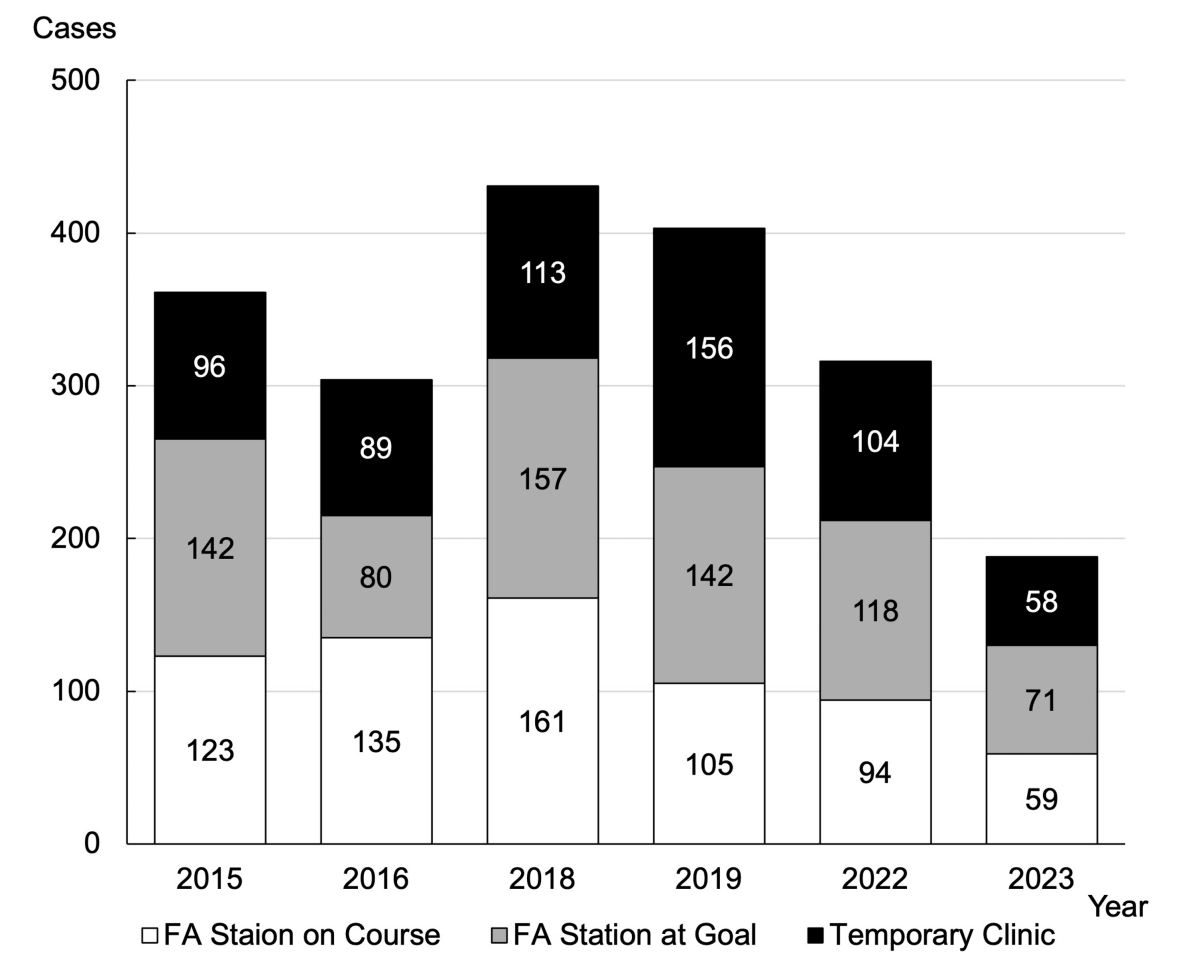
Number of injured or sick runners at the FA stations and temporary clinics The number of injured or sick runners has increased due to the change in the date in 2018, but due to improvements in the medical support system each year, the number of injured or sick runners being treated in each department is decreasing. FA - first aid; Goal - refers to the medical aid station located at the finish line

Figure 6 shows the breakdown of injured or sick runners at all the FA stations on the marathon course. In 2018, coinciding with warmer weather, the number of medical cases peaked (431 cases). In 2019, a scheme was launched to transport runners who needed medical intervention from the FA station on the course to temporary clinics as soon as possible, which led to an increase in the number of injured or sick runners treated at temporary clinics (156 runners, approximately 38.7% of the total 403 runners treated at temporary clinics). An analysis of patients requiring transport or hospitalization revealed that the majority were concentrated in the 40s and 50s age groups.

Table 1 presents a breakdown of the symptoms of injured or sick runners, including the number of emergency transports. Runners requiring medical intervention after emergency treatment were admitted to the hospital. Patients who required continued hospitalization were admitted because of severe dehydration (including heatstroke) and difficulty moving due to muscle cramps. Most patients needing transport or hospitalization were in their 40s or 50s.

**Table 1:** Breakdown of transport to hospital with symptoms

Year	Transport to hospital	Dehydration/ heat stroke	Nausea/ vomit	Seizure	Injury	Cardiac arrest	Muscle cramps	Unknown
									2015	9	4	1	0	0	0	4	0
2016	14	4	1	0	3	0	6	0
2018	55	25	10	7	3	1	6	3
2019	29	7	4	5	0	1	12	0
2022	20	8	1	1	5	1	4	0
2023	9	3	2	1	2	0	1	2

PPR and TTHR trends

**Figure 7:**
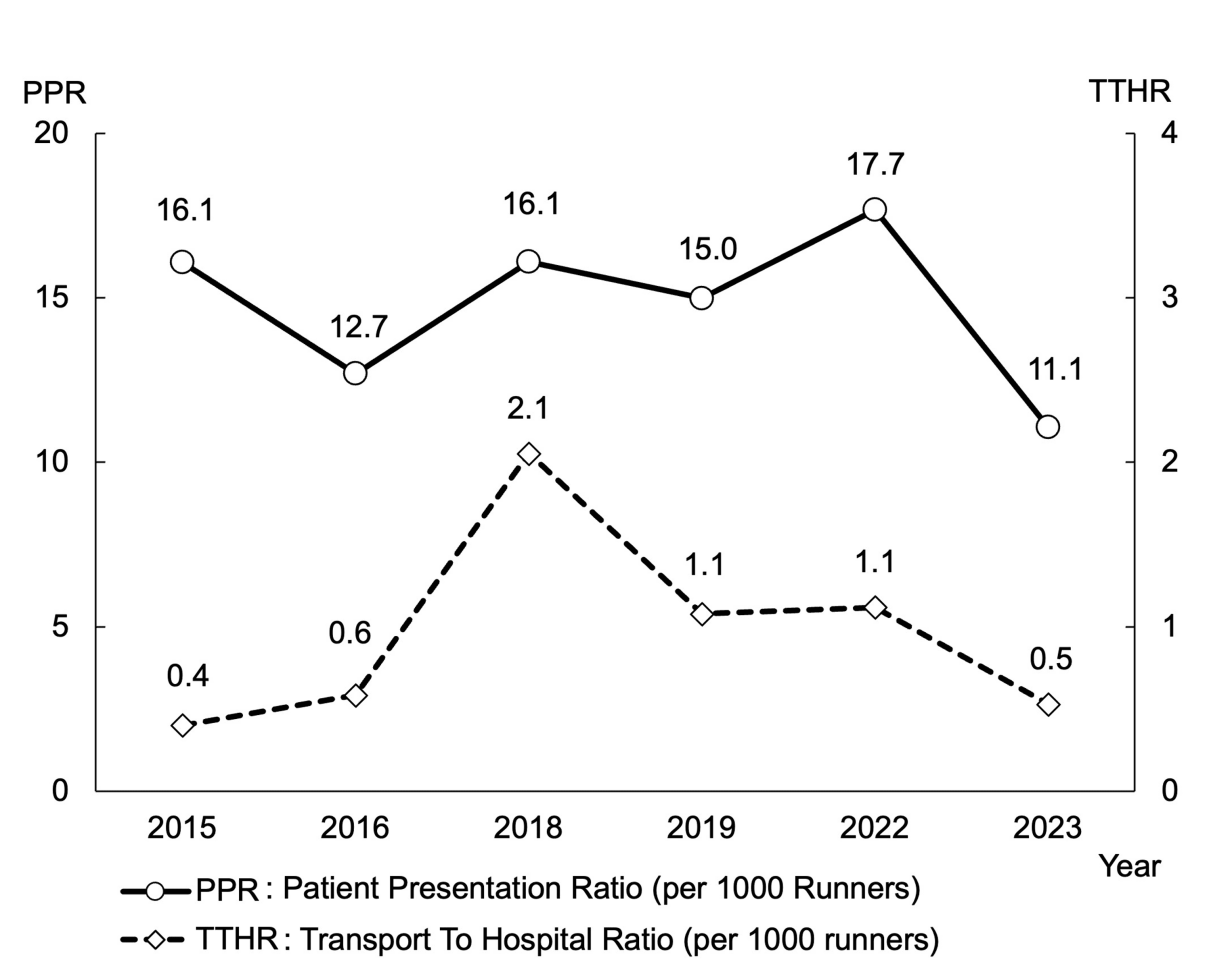
Change of the index of injured or sick runners and runners transferred to hospital The trend in patient presentation ratio (PPR), which indicates the prevalence of injured or sick, does not seem to have changed dramatically, but the transport-to-hospital ratio (TTHR), which indicates the rate of hospital transport, increased dramatically after the change in the date in 2018 and has since started to decrease. This downward trend may be due to improvements in the medical system through annual review.

Figure 7 shows the change in the index of injured and sick runners and emergency transport with PPR and TTHR. The PPR ranged from 12.7 to 17.7 per 1000 runners, showing minimal weather dependence. A slight rise in 2022 may reflect improved triage and referral. The TTHR peaked in 2018 (2.1) due to heatstroke but declined in later years (as low as 0.5) after countermeasures like increased hydration and better triage were implemented.

Cardiac arrest cases

Among the approximately 135,000 full marathon participants over the study period, three male runners experienced CA, resulting in an incidence rate of 2.2 per 100,000 runners (Table 2). Three male runners in their 40s-50s experienced cardiac arrest. All received immediate CPR, AED use within five minutes, and hospital care. All survived without long-term issues. However, limited expressway access slightly delayed transport in one case.

**Table 2:** Details of three runners with cardiac arrest CPR - cardiopulmonary resuscitation; AED - automated external defibrillator; ROSC - return of spontaneous circulation

	2018	2019	2022
Age/Sex	40s/Male	50s/male	40s/male
On set point	30 km (on highway）	4.2 km	30 km（on highway）
On set time	11:26 am	9:10 am	11:28 am
Time to start CPR	11:28 am	9:10 m	11:28 am
Time of AED attachment	11:31 am	9:14 am	11:32 am
Time of ROSC	11:33 am	9:20 am	11:32 am
Turning point	Return to society	Return to society	Return to society

## Discussion

In this study, we conducted a retrospective analysis of large-scale citizen marathons to investigate the incidence of injuries, illnesses, and CA among runners. A key finding was the observed trend of increased emergency transports with rising temperatures, suggesting a potential association with dehydration and heatstroke. However, improvements in the medical support system, such as enhanced triage, facilitated transport to temporary clinics, and the strategic deployment of BLS and FR teams, were found to be associated with observed reductions in emergency transports. The 'enhanced triage' implemented at the Yokohama Marathon involved several key improvements aimed at optimizing patient flow and prioritizing urgent cases. Specifically, this included: 1) standardized triage criteria: implementation of a clear, standardized triage protocol (e.g., based on vital signs, symptoms, and injury severity scales) for all medical staff at first aid stations, ensuring consistent assessment across the course. 2) Rapid assessment and referral: emphasis on rapid initial assessment to quickly identify critical cases requiring immediate transport to temporary clinics or hospitals rather than prolonged on-site treatment for severe conditions. 3) Dedicated triage personnel: in some years, dedicated medical personnel were assigned specifically to triage duties at high-volume stations to streamline the process. 4) Improved communication: enhanced communication channels between triage points and the central command center to facilitate timely decision-making and resource allocation for transport. While citizen marathons contribute to health promotion, it is also important to acknowledge that a full marathon of 42.195 km entails a significant physical burden for participants and is not suitable for everyone. Excessive training or inadequate preparation can increase the risk of injuries and other health issues.

The 'strategic deployment' of medical teams involved not only increasing their numbers but also optimizing their placement based on anticipated risk areas and historical data. This included: 1) high-risk zone prioritization: concentrating BLS/FR teams and first aid stations at segments of the course known for higher incidence of medical events, such as challenging uphill sections, congested areas, or later stages of the race where fatigue is prevalent. 2) Weather-adaptive adjustments: on days with predicted extreme weather (e.g., high temperatures or heavy rain), additional hydration stations were activated, and medical personnel were instructed to be particularly vigilant for heat-related illnesses or hypothermia. The deployment of mobile medical teams was also adjusted to provide more dynamic coverage. 3) Course-specific considerations: the Yokohama marathon course includes sections with varied terrain. What was previously referred to as 'steep banks' are more accurately described as steep slopes or inclines, particularly noticeable around the 30km mark or near the Bay Bridge ascent/descent. These sections demand greater physical exertion from runners and can contribute to musculoskeletal injuries or exertional heat stroke. Medical stations and mobile teams were strategically positioned before and after these challenging segments to provide immediate assistance. Compared to flatter marathon courses, the presence of these significant inclines necessitates a more robust and responsive medical presence in these specific areas.

In three CA cases, early CPR and AED usage within five minutes enabled all runners to survive and return to society. This outcome was confirmed through follow-up with the respective hospitals and the runners themselves, ensuring their full functional recovery and return to pre-event activities.

Environmental factors and health outcomes

The change in race timing from March to October 2018 led to higher temperatures and an increase in injured runners. Although no clear cutoff temperature predicting CA is established, ambulance dispatches are lowest at 22.5 °C and increase as temperatures deviate from this point, following a U-shaped curve [13]. Thus, ambient temperature appears to influence the incidence of injury or illness during races. In response to risks such as dehydration and heatstroke, we have annually increased water stations and aid points.

Faster runners are more affected by weather conditions than slower ones, with temperature having the greatest impact among variables such as humidity, wind speed, and solar radiation; women appear less susceptible than men. Interestingly, while performance may decline in rainy weather, finish times can improve [14].

The Wet Bulb Globe Temperature (WBGT), widely used to assess heat stress risk, was analyzed during the 2021 Tokyo Olympic marathon to evaluate potential heat impacts and countermeasures in the context of global warming [15]. The study recommended considering WBGT trends when determining the timing and location of future Summer Olympics. In our study, we measured WBGT annually; although values did not reach levels requiring activity restrictions, site-specific factors such as highway course exposure and variable conditions at the start and finish points may have contributed to injury and illness rates.

Specialized medical support system

Regarding the response of injured, sick, or runners with CA, CA is a major cause of death, with one in 7.4 individuals (approximately 13.5%) dying from CA, and 39% of adult cases being sports-related [16]. In marathons, the cardiac death rate ranges from 0.24 to 0.39 per 100,000 runners, with an incidence of 0.54 per 100,000 reported in one study [17, 18]. The incidence of CA in the Yokohama marathon was 2.2 per 100,000 runners. While this figure is numerically slightly higher than the reported incidence for the Tokyo Marathon (2.00 per 100,000 full marathon participants) [19], it is important to acknowledge that CA incidence in marathons can vary widely across different events and populations due to various factors including participant demographics, course characteristics, and environmental conditions. Given the descriptive nature of our study, we did not perform statistical comparisons to determine the significance of this difference. However, a highly commendable outcome of the Yokohama marathon's medical system, which is comparable to leading international marathon medical systems such as those in Boston, Chicago, and Berlin in terms of rapid response and favorable cardiac arrest outcomes, is that despite the observed CA events, there were no cardiac deaths reported throughout the 10-year study period. This highlights the effectiveness of the rapid response system, including immediate bystander CPR and prompt AED deployment, in achieving favorable outcomes for CA cases.

Key to successful resuscitation was minimizing the time from collapse to CPR and defibrillation. Large-scale events must ensure roadside medical teams can initiate CPR and deliver AED shocks promptly. Organizers should establish marathon-specific systems to enable immediate bystander CPR and defibrillation, with clear staff roles and communication protocols. 

**Figure 8:**
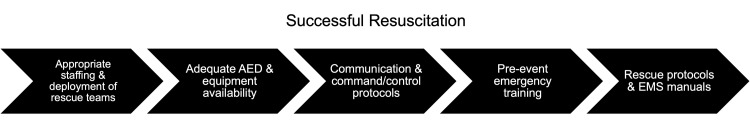
Five-pillar medical system for successful resuscitation Key factor of successful resuscitation for large-scale events with medical staff and volunteers. AED - automated external defibrillator; EMS - emergency medical services

To support this, we proposed a five-pillar medical system as Yokohama Marathon Medical Committee (Figure 8).

This system was shared with all staff, including volunteers, during pre-event briefings. Importantly, BLS and FR teams were positioned to initiate CPR within one minute and defibrillation within three minutes, using GPS for precise location tracking. Recognizing early signs, such as gasping, observed in 90.5% of CAs, is also crucial [20].

Medical operations and continuous review

We reviewed the previous year's issues using a medical rescue matrix and implemented improvements in the following year. In 2018, injuries and illnesses increased due to the date change (TTHR, 2.1), with dehydration and muscle cramps being major causes. Accordingly, we increased water stations. To reduce hospital transfers, we enhanced on-site triage and added protocols to direct runners needing medical care to temporary clinics (TTHR, 1.1). When the marathon resumed in 2022, external injuries rose (PPR, 17.7), possibly due to decreased exercise habits during the COVID-19 pandemic. Typical running injuries included muscle strains, sprains, and skin lesions, [11] often affecting the lower back and legs [21]. In 2023, FA stations expanded treatment areas and added massage spaces, leading to a lower PPR (11.1). This improvement was likely multifaceted, attributable not only to the enhanced facilities but also potentially to favorable weather conditions in 2023, which could have reduced the overall physiological strain on runners. The combined effect of proactive medical support system improvements, including refined triage and increased aid points, along with environmental factors and enhanced on-site care options like massage, collectively contributed to the observed reduction in patient presentation rates.

Overall, the PPR in our study (14.76, range, 11.1-17.7) was lower than in previous reports [22, 23], while the TTHR (0.96, range, 0.4-2.1) was similar or lower [22, 23]. This suggests the effectiveness of our medical management system, though outcomes may vary depending on race conditions. Notably, improvements were observed consistently in the same setting.

While the Yokohama Marathon Medical Committee conducts preliminary risk assessments, more detailed analyses are needed to identify underlying causes. These insights could inform injury prevention strategies through tailored training and optimized environments [24]. Given the multifactorial nature of running injuries, spanning personal, training, and lifestyle factors, prior risk evaluation, including weather variables such as WBGT, may help predict the incidence of injuries, illness, and cardiac arrest [24, 25].

Limitations

This study has some limitations. First, this study was retrospective, making it difficult to trace the details of injured, sick, and CA runners. Second, the backgrounds of injured, sick, or CA runners were based on self-reporting before the marathon race, which may not accurately reflect their actual conditions. Third, regarding the treatment of injured or sick runners, although the events were held in the same area, the medical staff was different annually; therefore, there may be slight differences in the provided treatment. Finally, the results reported in this study cannot be generalized to other marathon events, especially to races of different distances (e.g., half-marathons, 10K, 5K), or to other full marathons that may have different course designs, environmental conditions, event durations, or participant demographics. Importantly, as a descriptive study without inferential statistical analysis, we can only report observed associations and trends over time; we cannot establish causal relationships between changes in the medical support system and the observed outcomes.

## Conclusions

The Yokohama Citizen's Marathon's decade-long experience demonstrates that continuous evaluation and adaptive improvements in medical support are paramount for ensuring participant safety in large-scale mass-gathering events. Despite the prevalence of minor medical complaints, the successful resuscitation of all cardiac arrest cases highlights the critical importance of meticulous planning, robust interdisciplinary collaboration, and effective communication within the medical system. The "All Yokohama" coordinated medical system, characterized by its data-driven adjustments and strategic resource deployment, serves as a valuable model for other citizen marathons aiming to enhance the safety and well-being of their participants.
